# Research on Characteristics, Antioxidant and Antitumor Activities of Dihydroquercetin and Its Complexes

**DOI:** 10.3390/molecules23010020

**Published:** 2017-12-22

**Authors:** Yan Zhang, Juan Yu, Xiao-Dan Dong, Hai-Yu Ji

**Affiliations:** Key Laboratory of Food Nutrition and Safety, Ministry of Education, School of Food Engineering and Biotechnology, Tianjin University of Science and Technology, Tianjin 300457, China; haiyu11456@163.com (J.Y.); yujuan14615@163.com (X.-D.D.); jihaiyu1247@163.com (H.-Y.J.)

**Keywords:** dihydroquercetin complex, lecithin, β-cyclodextrin, physicochemical properties, antioxidant, antitumor activities

## Abstract

Dihydroquercetin is a kind of dihydroflavonol compounds with antioxidant, antitumor, antivirus and radioresistance activities. This study attempted to produce the dihydroquercetin complexes with lecithin and β-cyclodextrin, and research their characteristics and bioactivities via ultraviolet spectrum (UV), infrared spectroscopy (IR), scanning electron microscope (SEM), differential scanning calorimetry (DSC), X-ray diffraction spectrum (XRD), and MTT assay. Results showed that the complexes with lecithin and β-cyclodextrin could improve the solubility and dissolution rate, and remove the characteristic endothermic peak of dihydroquercetin. IR spectra proved their interaction, and results of SEM and XRD showed the amorphous characteristics of the dihydroquercetin compounds. These results indicated that dihydroquercetin was combined by lecithin or β-cyclodextrin with better physical and chemical properties, which would effectively improve the application value in the food and drug industries.

## 1. Introduction

Dihydroquercetin is a white crystalline pentahydroxy-flavanone commonly found in *Pseudotsuga taxifolia*, which is also known as taxifolin [1]. Dihydroquercetin exhibits various bioactivities—such as antitumor, antioxidant [2], and antivirus activities—and it also plays an important role in cardiovascular and liver diseases [3,4]. Therefore, dihydroquercetin has a high potential therapeutic promise to be developed as health food and pharmaceutical products, while its poor water-solubility limits the application.

Lecithin is a two-tail surfactant with a zwitterionic polar head and a negative phosphate group, which can self-assemble to form closed vesicles in proper conditions [5]. β-cyclodextrin is produced by the enzymatic degradation of starch and appears highly hydrophilic due to the presence of numerous hydroxyl groups [6]. Both lecithin and β-cyclodextrin are widely employed to enhance the stability, solubility, and bioavailability of guests for its lower price and higher production rate [7]. 

The aim of present study was to produce the dihydroquercetin complexes with lecithin and β-cyclodextrin, and research their characteristics, and antioxidant and antitumor activities. This work may provide a pathway to enhancing the water solubility and bioactivities of dihydroquercetin through preparing stable complexes.

## 2. Results

### 2.1. Solubility and Dissolution Rate 

Our results on solubility detection showed that the solubility of dihydroquercetin complexes with β-cyclodextrin and lecithin were 5.98 ± 0.02 mg/mL and 1.24 ± 0.02 mg/mL respectively, which were 24.9 and 5.17 times higher than that of dihydroquercetin (0.24 ± 0.01 mg/mL), suggesting that both β-cyclodextrin and lecithin could significantly increase the solubility of dihydroquercetin, and β-cyclodextrin exhibited a better improvement.

**Figure 1:**
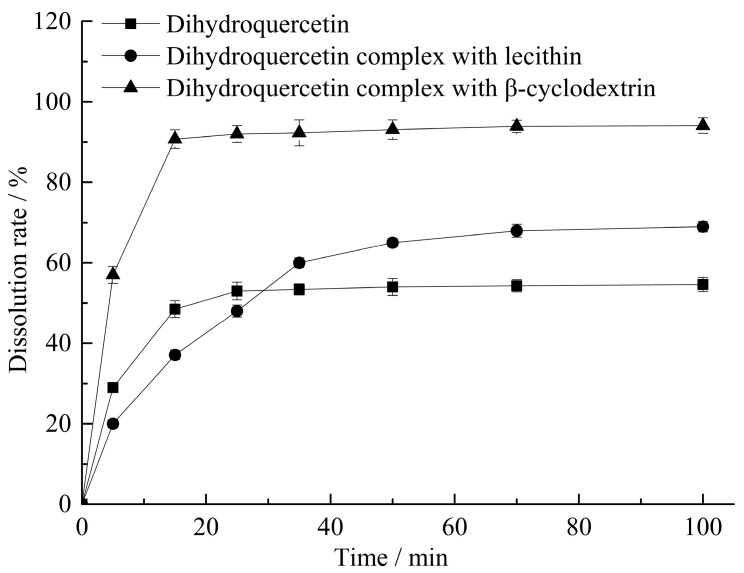
Dissolution rate detection.

The dissolution test results were showed in Figure 1, the cumulative dissolution rate of dihydroquercetin lecithin compound at 15 min was only 37.10%, while it would be gradually increased with the extended response time. The dissolution rate of dihydroquercetin complex with β-cyclodextrin was 90.76% at 15 min, which was 1.87 times higher than that of single dihydroquercetin. 

Phospholipids can make the complex viscous in water, which might interfere with the diffusion of dihydroquercetin, finally resulting in the lowest dissolution rate of the complex with lecithin in less than 25 min. β-cyclodextrin can form a hollow ring structure, which could embed in dihydroquercetin and enhance its water solubility.

### 2.2. UV and IR Analysis

**Figure 2:**
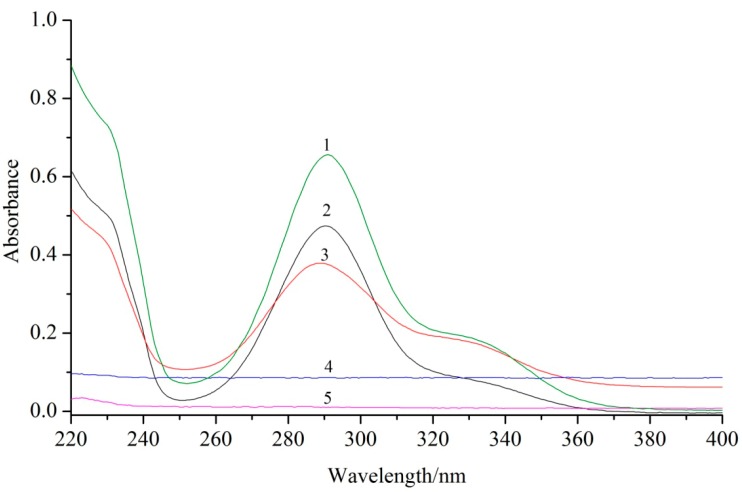
UV spectra analysis of dihydroquercetin (**1**), dihydroquercetin complex with lecithin (**2**), dihydroquercetin complex with β-cyclodextrin (**3**), β-cyclodextrin (**4**), and lecithin (**5**).

UV spectra analysis of dihydroquercetin and its complexes were shown in Figure 2, dihydroquercetin and the complexes with lecithin and β-cyclodextrin all exhibited a maximum absorption at 290 nm with similar basic shape, while the heights of these peaks were different, which indicated that the structure of the basic chromophoric group of dihydroquercetin was not changed in the process of complex formation. 

**Figure 3:**
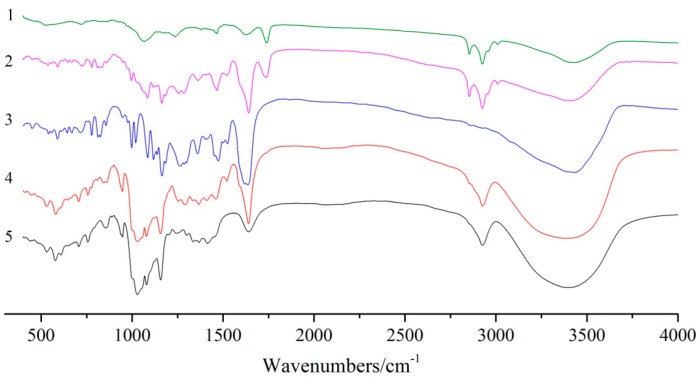
UV spectra analysis of lecithin (**1**), dihydroquercetin complex with lecithin (**2**), dihydroquercetin (**3**), dihydroquercetin complex with β-cyclodextrin (**4**), and β-cyclodextrin (**5**).

FT-IR spectroscopy is usually used to confirm the formation of inclusion complexes by investigating the variation of peaks. As shown in Figure 3, the FT-IR spectrum of soybean lecithin showed the prominent absorption bands at 2924 cm^−1^ (CH stretching vibration) and 1740 cm^−1^ (CO stretching vibration) [8]. The FT-IR spectrum of the dihydroquercetin complex with lecithin showed approximate superimposition of individual patterns of soybean lecithin and dihydroquercetin. For the FT-IR spectrum of β-cyclodextrin, a broad band at 3383 cm^−1^ was assigned to the symmetric and asymmetric OH stretching vibration due to the many intermolecular hydrogen bonds of β-cyclodextrin, and another bond at 2925 cm^−1^ represented CH stretching vibration, the absorption band at 1647 cm^−1^ was due to HOH bending [9]. These characteristic peaks of dihydroquercetin and β-cyclodextrin can also be found in the spectrum of dihydroquercetin complex with β-cyclodextrin, and no new absorption peaks were discovered, suggesting that no new covalent bonds were formed in dihydroquercetin β-cyclodextrin compound. 

### 2.3. Scanning Electron Microscope

**Figure 4:**
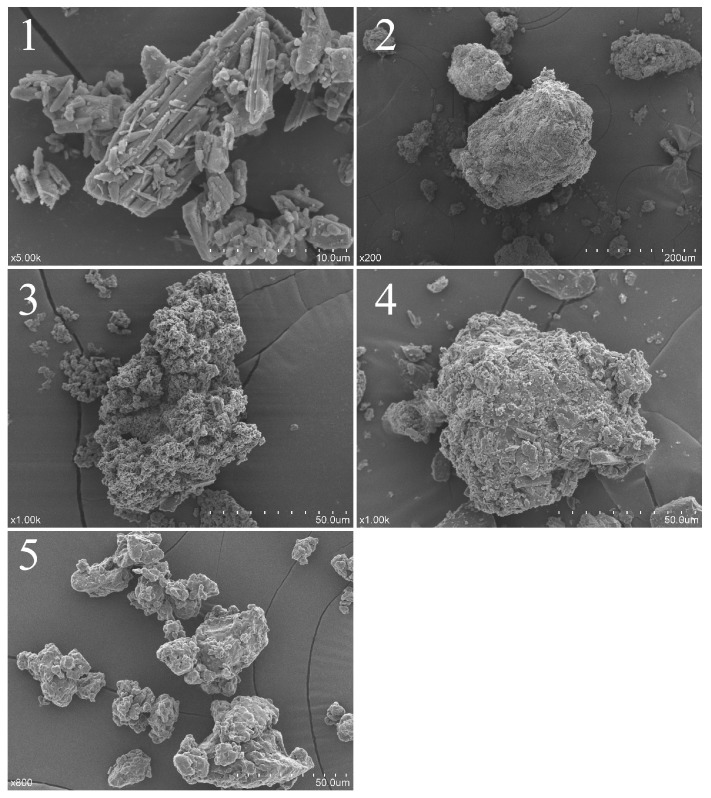
Scanning electron microscopy (SEM) analysis of dihydroquercetin (**1**), dihydroquercetin complex with lecithin (**2**), dihydroquercetin complex with β-cyclodextrin (**3**), β-cyclodextrin (**4**), and lecithin (**5**).

As shown in Figure 4, dihydroquercetin presented an acicular crystal form, lecithin showed an amorphous form and β-cyclodextrin exhibited a porous spherical structure, while dihydroquercetin complexes with lecithin and β-cyclodextrin showed a similar amorphous form with a larger scale, which indicated that dihydroquercetin seemed to be combined with lecithin and β-cyclodextrin, performed the appearance of the amorphous form, while the complex with lecithin showed a larger scale than that with β-cyclodextrin dimensionally.

### 2.4. DSC Analysis

**Figure 5:**
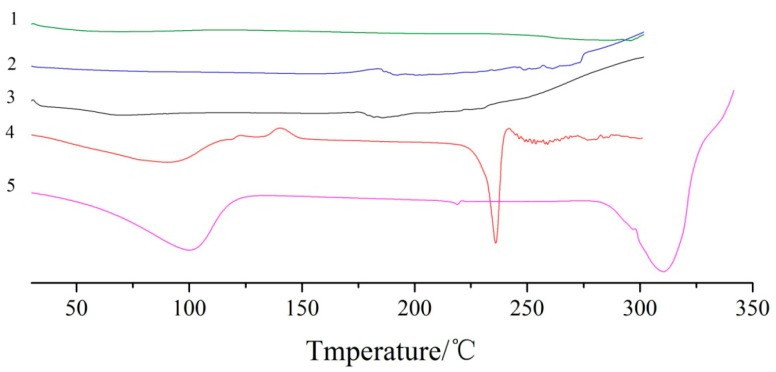
DSC curves of dihydroquercetin complex with β-cyclodextrin (**1**), lecithin (**2**), dihydroquercetin complex with lecithin (**3**), dihydroquercetin (**4**), and β-cyclodextrin (**5**).

DSC analysis of dihydroquercetin and its complexes were shown in Figure 5, dihydroquercetin began to melt at about 240 °C while lecithin showed no fixed melting point and no obvious heat absorption peak as a kind of amorphous substance, β-cyclodextrin had a broad peak at about 100 °C due to the loss of water and exhibited an endothermic peak at around 315 °C. Dihydroquercetin complexes showed no endothermic peaks, which indicated that lecithin and β-cyclodextrin formed a new phase with dihydroquercetin, resulting in the disappearance of their characteristic absorption peaks. 

### 2.5. XRD Analysis

**Figure 6:**
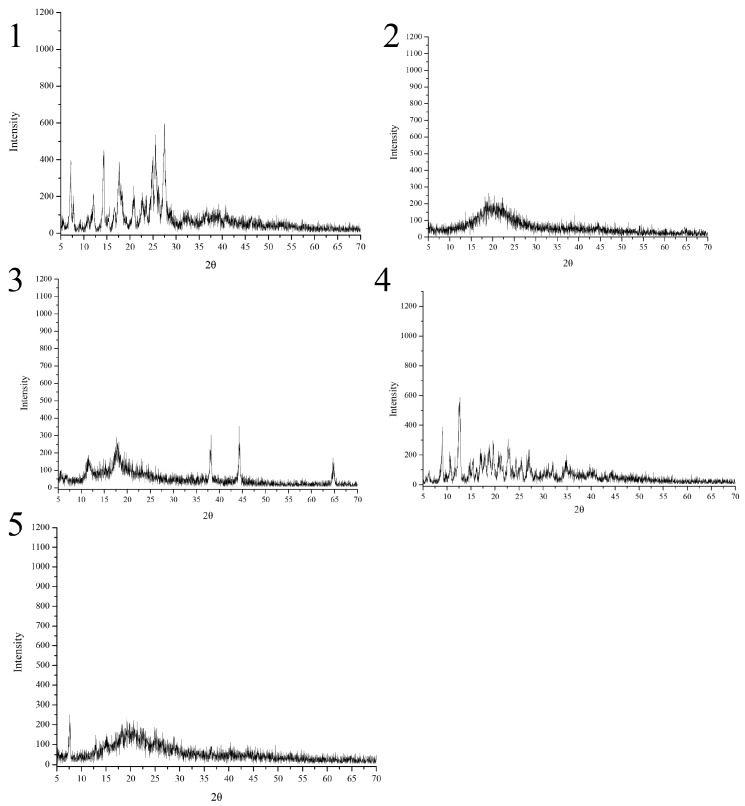
XRD spectra of dihydroquercetin (**1**), dihydroquercetin complex with lecithin (**2**), dihydroquercetin complex with β-cyclodextrin (**3**), β-cyclodextrin (**4**), and lecithin (**5**).

As shown in Figure 6, dihydroquercetin exhibited many crystalline peaks, indicating that dihydroquercetin mainly existed in a crystalline form, the spectrum of β-cyclodextrin showed that there no regular sequence of long atomic arrangement existed in crystal structure except a range of several atoms. Lecithin and its dihydroquercetin complex both showed amorphous characteristics according to the big broad peak [10]. The dihydroquercetin complex with β-cyclodextrin showed new diffraction peaks at 36°, 44°, and 65° (2θ), indicating the structural changes of dihydroquercetin and β-cyclodextrin when they formed an inclusion complex.

### 2.6. Antioxidant Activities In Vitro

**Figure 7:**
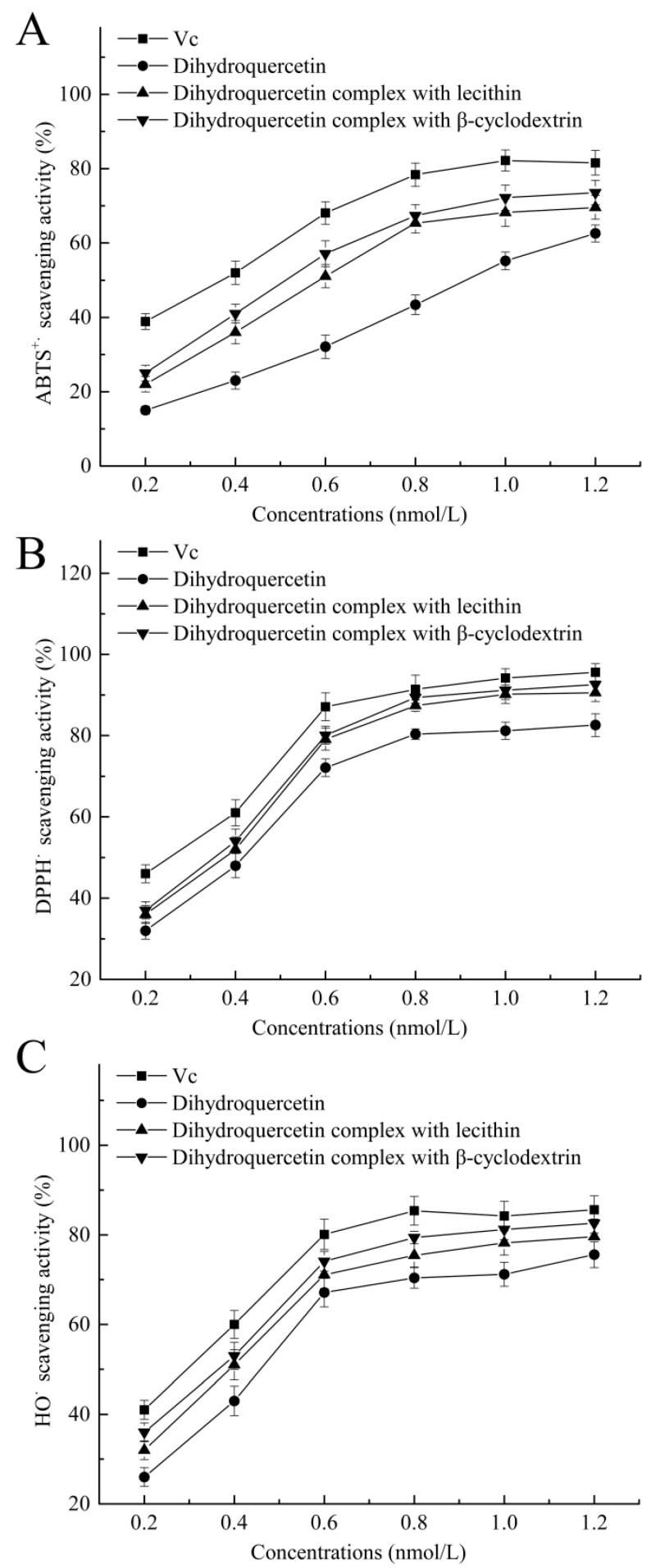
Antioxidant activities of dihydroquercetin and its complexes. (**A**) ABTS radical scavenging activity; (**B**) DPPH radical scavenging activity; (**C**) hydroxyl radical scavenging activity.

As shown in Figure 7, dihydroquercetin complex with β-cyclodextrin showed higher scavenging effects on ABTS^+·^ (72.2%), DPPH^·^ (91.2%), and HO^·^ (81.2%) at the concentration of 1 nmol/L, and dihydroquercetin complex with lecithin exhibited relatively lower scavenging effects on these free radicals. Dihydroquercetin showed the weakest radical scavenging activities due to the poor water solubility, while Vc was used as positive control and showed the highest scavenging rates on ABTS^+·^, DPPH^·^ and HO^·^ of 82.2, 94.2, and 84.2%, respectively. The results implied that β-cyclodextrin and lecithin were likely to improve the water solubility and antioxidant activity of dihydroquercetin, suggesting that the complexes could serve as free radical inhibitors or scavengers.

### 2.7. Antitumor Activity In Vitro

**Figure 8:**
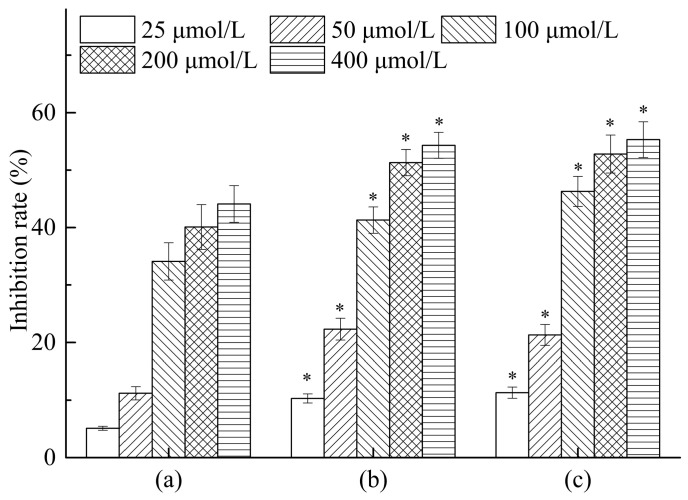
Antitumor activities of dihydroquercetin and its complexes. (**a**) Dihydroquercetin; (**b**) dihydroquercetin complex with lecithin; (**c**) dihydroquercetin complex with β-cyclodextrin. Note: * compare to dihydroquercetin at the same concentration, *p* < 0.05.

The results of in vitro antitumor activity of all compounds against HepG2 cells were summarized in Figure 8. Notably, dihydroquercetin showed good antitumor activity against HepG2 cell lines with an inhibition rate of 44.1% at a concentration of 400 μmol/L, while its complexes with lecithin and β-cyclodextrin both exhibited similar inhibition rates of 54.3% and 55.3% respectively, which were significantly increased compared with the dihydroquercetin only group. The results indicated that combining dihydroquercetin with β-cyclodextrin or lecithin could strengthen the in vitro antitumor activity of dihydroquercetin, suggesting that the complexes would possess a good potency for the treatment of human hepatocarcinoma.

## 3. Discussion

Dihydroquercetin has become a highly promising therapeutic substance for diseases such as cancer, oxidative cellular injury, cardiovascular disease, and liver disease [11,12]. However, its application is limited due to the poor water solubility. Lecithin is mainly obtained from soybeans and exhibits various bioactivities, including aging delay, regulation of blood lipid and cholesterol, and memory enhancement [13,14]. Cyclodextrin could be used as a crosslinker with a hydrophilic exterior, which could improve the water solubility of dihydroquercetin for better practical application [15,16]. The present study prepared the dihydroquercetin inclusion complexes with β-cyclodextrin and lecithin, which showed better water solubility and dissolution rate, especially the complex with β-cyclodextrin, which was consistent with the research by Yang et al. [17]. The structural characteristics analysis indicated that dihydroquercetin complex with lecithin showed no obvious endothermic or diffraction peaks with an amorphous form, while dihydroquercetin complex with β-cyclodextrin was globular and the endothermic and diffraction peaks were different from the individual patterns, indicating the formation of a novel phase.

Reactive oxygen species (ROS) were generated after the inflammatory phase, and could deleteriously damage DNA, proteins, and lipids, which would result in cell death and inactivation of innate antioxidant system [18]. DPPH is a stable free-radical compound which was widely used in assays to evaluate the ability of antioxidants to scavenge radicals [19]. The antioxidant response is primarily activated by preventing the generation of free radicals scavenging them directly, which may be the major contributor to the antioxidant mechanism [20]. It is also necessary to enhance the anticancer activity of existing materials since cancer has always been a major health problem for human beings with high rates of incidence and mortality [21]. Our results demonstrated that dihydroquercetin complex with β-cyclodextrin showed stronger antioxidant activity than the others, and the complexes with β-cyclodextrin and lecithin exhibited similarly higher in vitro antitumor activity against HepG2 cells, which indicated the importance and practicability of dihydroquercetin complexes with biomaterials.

## 4. Experimental Section

### 4.1. Materials and Chemicals

The sample of dihydroquercetin was provided by the Xi’an Natural Field Bio-technique Co. Ltd. (Xi’an, China). The β-cyclodextrin and lecithin were purchased from Sangon Biotech (Shanghai) Ltd. (Shanghai, China). Other reagents were analytical.

### 4.2. Preparation of Dihydroquercetin Complexes

100 mg dihydroquercetin and 200 mg of lecithin (400 mg of β-cyclodextrin) were added in 50 mL of tetrahydrofuran, and the supernatant was collected after magnetic stirring at room temperature for 4 h. The tetrahydrofuran was volatilized completely using nitrogen purging, and corresponding dihydroquercetin complexes were obtained after lyophilization. 

### 4.3. Solubility and Dissolution Rate Detection

Dihydroquercetin and its complexes with lecithin and β-cyclodextrin were respectively dissolved in distilled water at room temperature. After 5, 15, 25, 35, 50, 70, 100 min, 5 mL supernatant would be treated with 0.45 μm microporous membrane filtration and analyzed with HPLC, and the dissolution rate of dihydroquercetin in samples could be obtained. After 12 h, the supernatant would be treated with 0.45 μm microporous membrane filtration and analyzed with HPLC, and the solubility of dihydroquercetin in samples could be calculated.

### 4.4. UV and FT-IR Analysis

The UV–vis absorption spectra of dihydroquercetin and its complexes with lecithin and β-cyclodextrin, soybean lecithin, and β-cyclodextrin were recorded in the range from 220 to 400 nm to obtain the UV–vis absorption spectra by a scanning UV spectrophotometer (UV-2500PC, Shimadzu, Kyoto, Japan).

These powder samples were mixed with dry KBr in a ratio of 1:30 and pressed into a pellet, the analysis at the absorbance mode was conducted on a Fourier transformed IR spectrophotometer (Bruker VECTOR-22, Karlsruhe, Germany) with scanning range of 4000–400 cm^−1^ [22]. 

### 4.5. Scanning Electron Microscope (SEM)

A layer of gold was sputtered on dihydroquercetin and its complexes with lecithin and β-cyclodextrin, soybean lecithin, and β-cyclodextrin before testing, and the micrographs were obtained by a SU1510 scanning electronmicroscope (Hitachi, Tokyo, Japan) under 10 kV and low vacuum [23]. 

### 4.6. Differential Scanning Calorimetry (DSC)

Thermal analyses of dihydroquercetin and its complexes with lecithin and β-cyclodextrin, soybean lecithin, and β-cyclodextrin were performed by a DSC60 (Shimadzu, Japan). The temperature was increased at the speed of 10 °C/min from 30 °C to 300 °C under the protection of nitrogen, and the in-built software (TA-60WS, Shimadzu, Japan) was used to process the recorded data.

### 4.7. X-ray Powder Diffraction Spectra (XRD)

X-ray diffraction was conducted with a RU200R X-ray diffractometer (Shimadzu, Japan) with Cu Kα radiation at 40 kV and 40 mA, and the XRD spectra of dihydroquercetin and its complexes with lecithin and β-cyclodextrin, soybean lecithin, and β-cyclodextrin were recorded between 5° and 70° (2θ) with a scanning speed of 4 °C/min.

### 4.8. Antioxidant Activity In Vitro

#### 4.8.1. Total Antioxidant Activity Against ABTS Radicals

The antioxidant capacity against 2,2′-azino-bis(3-ethylbenzthiazoline-6-sulfonic acid) (ABTS) radicals was estimated following the modified procedure described previously [24]. Briefly, the ABTS solution was diluted with an ethanol: water (50:50) mixture to an absorbance of 0.70 ± 0.02 at 730 nm. After placing 20 µL sample of ascorbic acid (Vc) standard (6, 12, 18, 24, 30, 36 nmol/L) and 280 µL of diluted ABTS solution on a 96-well elisa plate, absorbance readings were taken at 730 nm after 20 min reaction. The scavenging activities of dihydroquercetin and its complexes were calculated by the following equation: 

Scavenging activity (%) = [1 − (A_1_ − A_2_)/A_0_] × 100, where A_1_ is the absorbance of the sample and ABTS solution, A_2_ is the absorbance of the sample and A_0_ is the absorbance of ABTS solution only. 

#### 4.8.2. DPPH Free Radical Scavenging Activity

The DPPH free radical scavenging activity was determined according to the previous research with some modifications [25]. Briefly, 2.0 mL DPPH methanol solution (0.08 mM) was added into 2.0 mL solution of dihydroquercetin and its complexes (0.4, 0.8, 1.2, 1.6, 2.0, and 2.4 nmol/L). The mixture was shaken thoroughly and incubated at room temperature for 30 min, and then determined at 517 nm. Ascorbic acid (Vc) was used as the positive control. The free radical scavenging activity was evaluated according to the following equation: 

Scavenging activity (%) = [1 − (A_1_ − A_2_)/A_0_] × 100, where A_1_ is the absorbance of the sample and DPPH, A_2_ is the absorbance of the sample only, and A_0_ is the absorbance of DPPH solution without sample. 

#### 4.8.3. Hydroxyl Radical (HO^·^) Scavenging Assay

The hydroxyl radical scavenging activity was assayed by the modified Fenton’s reaction as described previously [26]. The reaction mixture contained 1.0 mL of FeSO_4_, salicylic acid-ethanol, H_2_O_2_, and 1 mL different concentrations of sample solutions (0.4, 0.8, 1.2, 1.6, 2.0, 2.4 nmol/L). The solution was mixed thoroughly, and incubated at 37 °C for 30 min. The absorbance was determined at 510 nm. Vc was used as a positive control and distilled water was served as blank control. The scavenging activity of the hydroxyl radical was calculated as follows:

Scavenging activity (%) = [1 − (A_1_ − A_2_)/A_0_] × 100, where A_0_ is the absorbance of blank control, A_1_ is the absorbance of the sample in reactive system and A_2_ is the absorbance of the sample without HO^·^.

### 4.9. Antitumor Activity In Vitro

The inhibitory effects of samples on tumor cells were investigated using the colorimetric 3-(4,5-Dimethylthiazol-2-yl)-2,5-diphenyltetrazolium bromide (MTT) assay. The HepG2 cells (5 × 10^4^ cells/well) were inoculated into a 96-well plate for adhesion. After 24 h inoculation different concentrations (25, 50, 100, 200, and 400 μmol/L) of dihydroquercetin and its complexes were added and the mixtures were incubated at 37 °C and 5% CO_2_. After another 24 h the MTT reagent (5 mg/mL, 20 μL/well) was added for 3 h and the formazan crystals were dissolved by 150 μL of DMSO. The optical density (OD) values were measured for each well at 490 nm. The antitumor activity was expressed as inhibition rate calculated as the following formula:

Inhibitory rate (%) = (OD_1_ − OD_0_)/OD_1_ × 100, where OD_1_ and OD_0_ represent the absorbance of the control and sample respectively. 

### 4.10. Statistical Analysis

All values were presented as the mean ± standard deviation (S.D.). Statistical analyses of these data were performed with SPSS 19.0. The significance of difference was analyzed with one-way analysis of variance (ANOVA). A value of *p* < 0.05 was considered statistically significant.

## 5. Conclusions 

Dihydroquercetin complexes with lecithin and β-cyclodextrin were successfully prepared and their characteristics and bioactivities were analyzed via UV, FT-IR, SEM, DSC, XRD, and MTT assay. By forming inclusion complexes, the solubility and dissolution rate in water were significantly improved and different physicochemical characteristics were formed compared to individual patterns. More importantly, the complex exhibited stronger antioxidant and antitumor activities in vitro, especially the complex with β-cyclodextrin. Our study proved that dihydroquercetin complexes had the potential for improving application values in the food and medicine industries.
